# Supplementary material for: Understanding the neurodynamic process of decision-making for mobile application downloading
Source: PLoS One. 2022 Dec 7;17(12):e0278753. doi: 10.1371/journal.pone.0278753 (PMC9728891; doi:10.1371/journal.pone.0278753)
Supplement: S1 File — (DOCX) [file pone.0278753.s001.docx]

**Understanding the Neurodynamic Process of Decision-Making for Mobile Application Downloading.**

Harshit Parmar^1^, Fred Davis^2^ and Eric Walden^2*^

^1^ Texas Tech Neuroimaging Institute, Texas Tech University, Lubbock, Texas, USA

^2^ Rawls College of Business, Texas Tech University, Lubbock, Texas, USA

***** Corresponding author

E-mail: eric.walden@ttu.edu

The authors contributed equally to this work.

Understanding the Neurodynamic Process of Decision-Making for Mobile Application Downloading.

***Supplementary Material***

# Appendix 1: Independent Components

The details about all the independent components (ICs) are given in Appendix 1. Table S1 below shows the dynamic range and the fALFF score for all ICs. Dynamic range is the difference between the peak power and minimum power at frequencies to the right of the peak. The fALFF score is the low frequency to high frequency power ratio. Figure S1 below shows the spatial maps corresponding to all 50 ICs with orthogonal slices displayed at the peak voxel location. The color bar represents the normalized ICA score. Voxels greater than a z-score (normalized ICA score) of 1 are shown in the image.

Table S1: Dynamic range and fALFF score for all 50 IC

| **IC #** | **Dynamic Range** | **fALFF score** |  | **IC #** | **Dynamic Range** | **fALFF score** |
| --- | --- | --- | --- | --- | --- | --- |
| *1* | 0.046 | 8.099 |  | *26* | 0.047 | 2.619 |
| *2* | 0.052 | 8.341 |  | *27* | 0.045 | 4.209 |
| *3* | 0.023 | 0.468 |  | *28* | 0.021 | 0.746 |
| *4* | 0.050 | 6.490 |  | *29* | 0.052 | 7.204 |
| *5* | 0.041 | 4.201 |  | *30* | 0.023 | 0.483 |
| *6* | 0.048 | 11.167 |  | *31* | 0.047 | 5.262 |
| *7* | 0.045 | 5.216 |  | *32* | 0.023 | 0.323 |
| *8* | 0.034 | 2.010 |  | *33* | 0.025 | 0.647 |
| *9* | 0.033 | 1.516 |  | *34* | 0.049 | 6.528 |
| *10* | 0.020 | 0.666 |  | *35* | 0.018 | 0.502 |
| *11* | 0.047 | 4.066 |  | *36* | 0.055 | 24.733 |
| *12* | 0.032 | 1.822 |  | *37* | 0.050 | 15.451 |
| *13* | 0.037 | 1.136 |  | *38* | 0.048 | 3.370 |
| *14* | 0.045 | 4.792 |  | *39* | 0.026 | 0.971 |
| *15* | 0.038 | 1.071 |  | *40* | 0.047 | 5.802 |
| *16* | 0.021 | 0.632 |  | *41* | 0.030 | 0.595 |
| *17* | 0.042 | 4.452 |  | *42* | 0.028 | 1.702 |
| *18* | 0.050 | 4.228 |  | *43* | 0.026 | 0.482 |
| *19* | 0.028 | 1.412 |  | *44* | 0.023 | 0.635 |
| *20* | 0.027 | 0.778 |  | *45* | 0.041 | 1.607 |
| *21* | 0.019 | 0.599 |  | *46* | 0.021 | 0.746 |
| *22* | 0.026 | 0.785 |  | *47* | 0.053 | 5.959 |
| *23* | 0.071 | 22.617 |  | *48* | 0.049 | 5.152 |
| *24* | 0.021 | 0.633 |  | *49* | 0.021 | 0.656 |
| *25* | 0.050 | 11.083 |  | *50* | 0.038 | 2.048 |


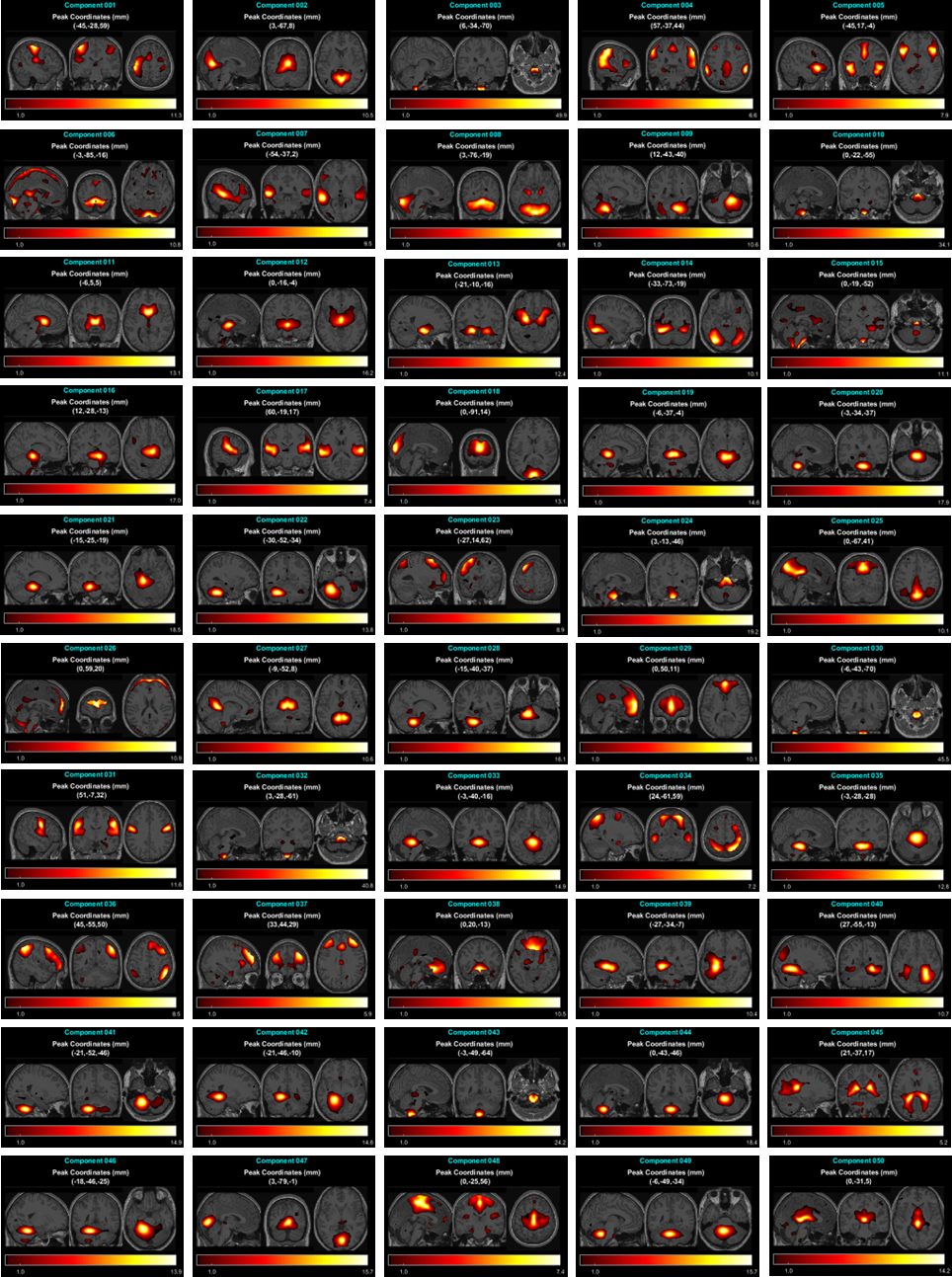


Figure S1: Spatial maps for all 50 ICs. The image shows orthogonal slices at the peak voxel whose coordinates are indicated above.

# Appendix 2: Cluster level details for the GLM Analysis

The details about GLM analysis are shown in this section. Table S2 below shows the cluster level information for (a) *YES > NO*, (b) *NO > YES* and (c) *Both > Baseline* contrasts. The table shows the family-wise error (FWE) probability (pFWE), total number of voxels in a cluster (k), peak t-statistics value (T) and the location (MNI coordinates) of the peak voxel.

Table S2: Cluster level information for (a) YES > NO, (b) NO > YES and (c) Both > Baseline contrast.

| ***YES > NO*** | | | | | |
| --- | --- | --- | --- | --- | --- |
| **pFWE** | **k** | **T** | **Peak MNI coordinates** | | |
|  |  |  | **X** | **Y** | **Z** |
| <0.001 | 14 | 6.08 | 54 | 38 | -4 |
| 0.004 | 11 | 5.34 | -51 | 23 | -4 |
|  |  | (a) |  |  |  |
|  |  |  |  |  |  |
| ***NO > YES*** | | | | | |
| **pFWE** | **k** | **T** | **Peak MNI coordinates** | | |
|  |  |  | **X** | **Y** | **Z** |
| <0.001 | 85 | 6.47 | -3 | -82 | 20 |
| 0.002 | 52 | 5.51 | -6 | -76 | 56 |
|  |  | 5.12 | 9 | -76 | 56 |
| 0.006 | 17 | 5.28 | 0 | -55 | 62 |
| 0.016 | 15 | 5.05 | 27 | -46 | 71 |
|  |  | (b) |  |  |  |
|  |  |  |  |  |  |
| ***BOTH > Baseline*** | | | | | |
| **pFWE** | **k** | **T** | **Peak MNI coordinates** | | |
|  |  |  | **X** | **Y** | **Z** |
| <0.001 | 8853 | 22.67 | -3 | -85 | 23 |
|  |  | 22.51 | 15 | -79 | 56 |
|  |  | 18.99 | 12 | -85 | 35 |
| <0.001 | 180 | 9.69 | 42 | 5 | 44 |
|  |  | 8.21 | 42 | 2 | 65 |
| <0.001 | 31 | 7.96 | 45 | 35 | 20 |
| <0.001 | 42 | 7.92 | 48 | 14 | 23 |
|  |  | (c) |  |  |  |

# Appendix 3: Unthresholded Spatiotemporal Activation

The unthresholded spatiotemporal activation plot is shown in Figure S2 below. The color indicates the number of subjects which showed activation for a given time-ROI pair.


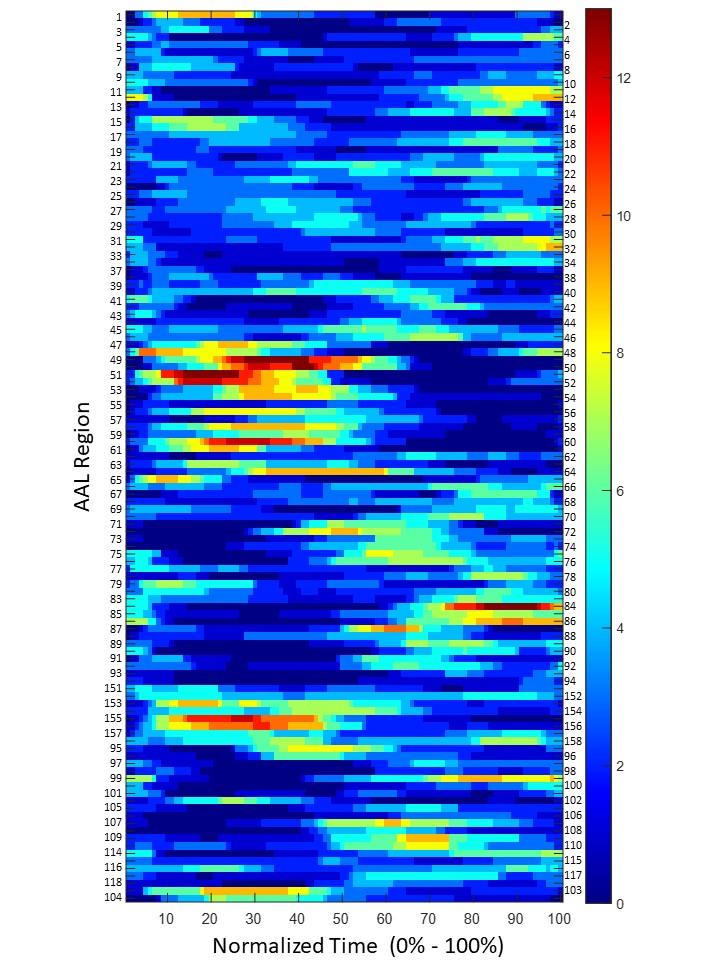


Figure S2: Unthresholded spatiotemporal activation plot for all AAL regions. The AAL region numbers are indicated on the Y-axis while the normalized time is indicated on the X-axis. The color indicates the number of subjects for which significant activation is observed in each time window – ROI pair.

# Appendix 4: AAL atlas regions

The different brain regions corresponding to the AAL ROI number is shown in Table S3 below.

Table S3: AAL atlas ROI number and corresponding brain region.

| **AAL** | **ROI name** |  | **AAL** | **ROI name** |  | **AAL** | **ROI name** |
| --- | --- | --- | --- | --- | --- | --- | --- |
| 1 | Precentral_L |  | 31 | OFClat_L |  | 61 | Postcentral_L |
| 2 | Precentral_R |  | 32 | OFClat_R |  | 62 | Postcentral_R |
| 3 | Frontal_Sup_2_L |  | 33 | Insula_L |  | 63 | Parietal_Sup_L |
| 4 | Frontal_Sup_2_R |  | 34 | Insula_R |  | 64 | Parietal_Sup_R |
| 5 | Frontal_Mid_2_L |  | 35 | Cingulate_Ant_L |  | 65 | Parietal_Inf_L |
| 6 | Frontal_Mid_2_R |  | 36 | Cingulate_Ant_R |  | 66 | Parietal_Inf_R |
| 7 | Frontal_Inf_Oper_L |  | 37 | Cingulate_Mid_L |  | 67 | SupraMarginal_L |
| 8 | Frontal_Inf_Oper_R |  | 38 | Cingulate_Mid_R |  | 68 | SupraMarginal_R |
| 9 | Frontal_Inf_Tri_L |  | 39 | Cingulate_Post_L |  | 69 | Angular_L |
| 10 | Frontal_Inf_Tri_R |  | 40 | Cingulate_Post_R |  | 70 | Angular_R |
| 11 | Frontal_Inf_Orb_2_L |  | 41 | Hippocampus_L |  | 71 | Precuneus_L |
| 12 | Frontal_Inf_Orb_2_R |  | 42 | Hippocampus_R |  | 72 | Precuneus_R |
| 13 | Rolandic_Oper_L |  | 43 | ParaHippocampal_L |  | 73 | Paracentral_Lobule_L |
| 14 | Rolandic_Oper_R |  | 44 | ParaHippocampal_R |  | 74 | Paracentral_Lobule_R |
| 15 | Supp_Motor_Area_L |  | 45 | Amygdala_L |  | 75 | Caudate_L |
| 16 | Supp_Motor_Area_R |  | 46 | Amygdala_R |  | 76 | Caudate_R |
| 17 | Olfactory_L |  | 47 | Calcarine_L |  | 77 | Putamen_L |
| 18 | Olfactory_R |  | 48 | Calcarine_R |  | 78 | Putamen_R |
| 19 | Frontal_Sup_Medial_L |  | 49 | Cuneus_L |  | 79 | Pallidum_L |
| 20 | Frontal_Sup_Medial_R |  | 50 | Cuneus_R |  | 80 | Pallidum_R |
| 21 | Frontal_Med_Orb_L |  | 51 | Lingual_L |  | 81 | Thalamus_L |
| 22 | Frontal_Med_Orb_R |  | 52 | Lingual_R |  | 82 | Thalamus_R |
| 23 | Rectus_L |  | 53 | Occipital_Sup_L |  | 83 | Heschl_L |
| 24 | Rectus_R |  | 54 | Occipital_Sup_R |  | 84 | Heschl_R |
| 25 | OFCmed_L |  | 55 | Occipital_Mid_L |  | 85 | Temporal_Sup_L |
| 26 | OFCmed_R |  | 56 | Occipital_Mid_R |  | 86 | Temporal_Sup_R |
| 27 | OFCant_L |  | 57 | Occipital_Inf_L |  | 87 | Temporal_Pole_Sup_L |
| 28 | OFCant_R |  | 58 | Occipital_Inf_R |  | 88 | Temporal_Pole_Sup_R |
| 29 | OFCpost_L |  | 59 | Fusiform_L |  | 89 | Temporal_Mid_L |
| 30 | OFCpost_R |  | 60 | Fusiform_R |  | 90 | Temporal_Mid_R |

Table S3 (cont.): AAL atlas ROI number and corresponding brain region.

| **AAL** | **ROI name** |  | **AAL** | **ROI name** |  | **AAL** | **ROI name** |
| --- | --- | --- | --- | --- | --- | --- | --- |
| 91 | Temporal_Pole_Mid_L |  | 118 | Vermis_8 |  | 145 | Thal_PuM_L |
| 92 | Temporal_Pole_Mid_R |  | 119 | Vermis_9 |  | 146 | Thal_PuM_R |
| 93 | Temporal_Inf_L |  | 120 | Vermis_10 |  | 147 | Thal_PuA_L |
| 94 | Temporal_Inf_R |  | 121 | Thal_AV_L |  | 148 | Thal_PuA_R |
| 95 | Cerebellum_Crus1_L |  | 122 | Thal_AV_R |  | 149 | Thal_PuL_L |
| 96 | Cerebellum_Crus1_R |  | 123 | Thal_LP_L |  | 150 | Thal_PuL_R |
| 97 | Cerebellum_Crus2_L |  | 124 | Thal_LP_R |  | 151 | ACC_sub_L |
| 98 | Cerebellum_Crus2_R |  | 125 | Thal_VA_L |  | 152 | ACC_sub_R |
| 99 | Cerebellum_3_L |  | 126 | Thal_VA_R |  | 153 | ACC_pre_L |
| 100 | Cerebellum_3_R |  | 127 | Thal_VL_L |  | 154 | ACC_pre_R |
| 101 | Cerebellum_4_5_L |  | 128 | Thal_VL_R |  | 155 | ACC_sup_L |
| 102 | Cerebellum_4_5_R |  | 129 | Thal_VPL_L |  | 156 | ACC_sup_R |
| 103 | Cerebellum_6_L |  | 130 | Thal_VPL_R |  | 157 | N_Acc_L |
| 104 | Cerebellum_6_R |  | 131 | Thal_IL_L |  | 158 | N_Acc_R |
| 105 | Cerebellum_7b_L |  | 132 | Thal_IL_R |  | 159 | VTA_L |
| 106 | Cerebellum_7b_R |  | 133 | Thal_Re_L |  | 160 | VTA_R |
| 107 | Cerebellum_8_L |  | 134 | Thal_Re_R |  | 161 | SN_pc_L |
| 108 | Cerebellum_8_R |  | 135 | Thal_MDm_L |  | 162 | SN_pc_R |
| 109 | Cerebellum_9_L |  | 136 | Thal_MDm_R |  | 163 | SN_pr_L |
| 110 | Cerebellum_9_R |  | 137 | Thal_MDl_L |  | 164 | SN_pr_R |
| 111 | Cerebellum_10_L |  | 138 | Thal_MDl_R |  | 165 | Red_N_L |
| 112 | Cerebellum_10_R |  | 139 | Thal_LGN_L |  | 166 | Red_N_R |
| 113 | Vermis_1_2 |  | 140 | Thal_LGN_R |  | 167 | LC_L |
| 114 | Vermis_3 |  | 141 | Thal_MGN_L |  | 168 | LC_R |
| 115 | Vermis_4_5 |  | 142 | Thal_MGN_R |  | 169 | Raphe_D |
| 116 | Vermis_6 |  | 143 | Thal_PuI_L |  | 170 | Raphe_M |
| 117 | Vermis_7 |  | 144 | Thal_PuI_R |  |  |  |

# Appendix 5: Machine Learning Classification

The features from representative time series of select AAL brain regions were used to train a machine learning model to predict the download decision of the participant. The neural network consists of one hidden layer with 32 nodes. As described in the results section, 10 AAL regions were identified from the spatiotemporal analysis of YES and NO apps. The regions are amygdala (AAL 69), ACC (AAL 151), left angular gyrus (AAL 69), posterior DMN (AAL71, 72), cerebellum (AAL 109, 117), DLPFC (AAL 5), VLPFC (AAL 11) and VMPFC (AAL 21). A pairwise correlation was computed between the time series of each of the 10 regions for all the apps. The pairwise correlation values were stored in a 10x10 matrix. Because the correlation matrix is symmetric, only the 45 unique values from the lower triangular matrix were considered as input.

Such 45-dimensional input vector was computed for all apps and all participants. The data was the split into 5 equal numbered portions. At any time, 4 of those were considered for training while the remaining partition was considered for testing. The neural network was trained using SGDM (stochastic gradient descent with momentum) algorithm using MATLAB. The results of the 5-fold cross validation are given in the results section.
