# Supplementary material for: Understanding the neurodynamic process of decision-making for mobile application downloading
Source: PLoS One. 2022 Dec 7;17(12):e0278753. doi: 10.1371/journal.pone.0278753 (PMC9728891; doi:10.1371/journal.pone.0278753)
Supplement: Attachment — Submitted filename: PLOS ONE-Review2208_HL.docx [file pone.0278753.s002.docx]

**Review for PLOS ONE, PONE-D-21-41018**

**Title:**	Understanding the Neurodynamic Process of Decision-Making for Mobile Application Downloading.

**Authors:** Harshit Parmar, Fred Davis and Eric Walden

**Reviewer**: Hans Liljenström

**COMMENTS** on the paper entitled, ” Understanding the Neurodynamic Process of Decision-Making for Mobile Application Downloading”, submitted to PLOS ONE in July 2022

**General comments**

This paper is about an exploration of the decision making process during fictive mobile app downloading, as a proxy for technology acceptance/adoption (TA). The aim is to suggest a neural model for the different consequtive phases in the decision making (DM) process. The paper is clearly written, carefully explaining the methodology and results. There is also a section with “challenges and limitations”, where the authors, in an insightful way, describe what they perceive are difficulties with their study, and where more research is called for.

My main concern with this paper is that the fictive app download is quite artificial, and may say little about the decision making process in the brain, considering a realistic situation when choosing apps to download. The decision to download an app or not may be considered arbitrary in some paradigms, since there is no experimental reward (i.e. skin in the game) provided as in e.g. (Maoz et al., 2019). This may weaken possible emotional or reward responses. While reflecting decision-making on app downloading, generally disinterest in the app (decision to act) or daydreaming may be more correlated with ‘No’ and may also drive a less salient decision. The “default mode” towards a ‘Yes’ could just reflect an inclination to choose more or less all apps, which are not perceived as bad or totally useless. If a cost of some sort (e.g. a limited budget or storage space).

It is also questionable if the fictive app download could actually be considered a TA, as there is no testing of the apps, and it is not clear what the Yes or No implies, if the subjects have understood the apps, and think they may be useful, or whether they just base their “decisions” on look, on mood, or plainly randomly. The reasons for answering Yes or No may be vague and unclear, also given the compressed description of the apps during the experiment.

While the results and conclusions made are not totally convincing, the paper is a good attempt to trace the different steps in the decision making process of the brain, and thus opens up for further studies of this sort.

**Specific comments and questions**

When the authors talk about neurodynamics, it should be noted that it is macroscopic neurodynamics, and not the mesoscopic neurodynamics perhaps mostly used with EEG in cognitive tasks. As the authors recognize, fMRI has a rather low temporal resolution in the order of seconds, which cannot capture the fast dynamics in many decision making cases (c.f. the Libet type of experiments on volition). Hence, it is difficult to trace causal pathways involving neural events or processes at faster time scales.

It is clear and expected to see initial activation in sensory (visual) and attention areas, but it is less clear where and when any decision is taken, even though PFC areas are involved.

Dynamics were observed in the cerebellum and amygdala but apparently not in the thalamus or basal ganglia. How about the SMA or pre-SMA, which have been reported to play an important role in decision making and volition? Why are these areas not included or displayed in the study?

The omission of resting states between choices is a bit problematic, as also the authors recognize. It seems quite clear that there will be some overlap/leakage between the activity of previous and the following app presentation/decision, and this may distort the activation patterns considerably. Perhaps the advantage with resting state periods exceeds the disadvantages? It could be worth comparing both methods.

There seems to be a rather low number of (eligible) subjects for the experiment, making any statistical analysis problematic, and conclusions questionable. It is also not stated whether all those subjects were right-handed, which may have an effect on the activity in motor cortices.

The types of apps are not reported, neither there attractiveness for various purposes, nor the (fictive or real) prices, which all may influence the choices by the subjects. The familiarity or previous experience with the same or similar apps may drastically affect the decision and decision time. It would have been good if the authors had displayed the apps used in the experiments, and preferably also the subjects preferences/history with regard to similar apps.

Data and other material is referred to an Appendix, which appears to be missing.

GLM, as other acronyms or abreviations, should be spelled out the first time it is mentioned.

The machine learning method used for prediction is not explained or described, which would have been desired, perhaps in an appendix.

The conclusions are a bit vague and not totally based on the presented results. That the largest activation is in the visual cortex is not surprising, and is likely not significant for the decision making process. Instead, it is probably just reflecting the reading/perception of the app description. It could have been good to compare the activity in a situation without a decision following the visual presentation of the app. The authors also claim the decision can be predicted toward the middle of the process, but that is not really demonstrated.

**Figures and tables**

The figures are in general good and descriptive. However, some of the figures are confusing or hard to understand:

Fig. 2 has an insufficient legend.

In Fig. 3, Yes>No might represent activation of language centers in VLPFC, possibility indicating a more careful read of the description. But, this may not represent the actual decision making. One of two activated regions for No>Yes wasn't mentioned. It looks like visual cortex, perhaps V3 or MT but between hemispheres. Is this an artifact?

In Fig. 6 it is difficult to see or follow temporal/causal pathways, and in particular where the decision is taking place. It also seems that the (left) motor cortex peaks about 4.4 secs after the button press, which may be an effect of the BOLD delay, but should be explained.

In Fig. 10, the different boxes represent different brain regions, according to the legend, but in fact there is a confusing mixture of brain regions and functions in the figure.

A reference table with the used AAL codes and brain areas might help interpretation.

**References**

The authors are suggested to consider any or all of the following articles for further references:

1. Frank, M.J. Claus, E.D. (2006) Anatomy of a decision: striato-orbitofrontal interactions in reinforcement learning, decision making, and reversal. *Psychological Review*. 113(2):300-26.
2. Hassannejad Nazir, A. & Liljenström, H. (2015). A cortical network model of cognitive and emotional influences in human decision making. *Biosystems* 136:128-141. [doi.org/10.1016/j.biosystems.2015.07.004](https://doi.org/10.1016/j.biosystems.2015.07.004)
3. Levine, D.S (2012) Neural dynamics of affect, gist, probability, and choice. *Cognitive Systems Research.* Volume 15-16 : 57-72.
4. Maoz U, Yaffe G, Koch C, Mudrik L (2019) Neural precursors of deliberate and arbitrary decisions in the study of voluntary action. Elife 8:e39787. https:// doi. org/ 10. 7554/ eLife. 39787. 001
